# EGFR is involved in dermatofibrosarcoma protuberans progression to high grade sarcoma

**DOI:** 10.18632/oncotarget.23899

**Published:** 2018-01-03

**Authors:** Amélie Osio, Shuo Xu, Morad El Bouchtaoui, Christophe Leboeuf, Guillaume Gapihan, Christine Lemaignan, Guilhem Bousquet, Céleste Lebbé, Anne Janin, Maxime Battistella

**Affiliations:** ^1^ Pathology Department, Hôpital St Louis, APHP, Paris, France; ^2^ Université Paris Diderot, Inserm, UMR_S1165, Paris, France; ^3^ Oncology Department, Hôpital St Louis, APHP, Paris, France; ^4^ Oncology Department, Hôpital Avicenne, Bobigny, France; ^5^ Université Paris 13, Villetaneuse, France; ^6^ Dermatology Department, Hôpital St Louis, Paris, France; ^7^ Université Paris Diderot, Inserm, UMR_S976, Paris, France

**Keywords:** soft tissue sarcoma, dermatofibrosarcoma protuberans, tumor heterogeneity, EGFR, SNAIL

## Abstract

Dermatofibrosarcoma protuberans (DFSP), amounting to 6% of all soft tissue sarcomas, has a slow growth rate, contrasting with a likelihood for local recurrence and a 10-20% evolution to higher-grade sarcoma, or “transformed DFSP” (DFSP-T). At molecular level, the characteristic COL1A1-PDGFB rearrangement, leading to sustained PDGFR signaling, is not linked to the evolutive potential. Here, we studied EGFR, another tyrosine kinase receptor, using laser-microdissection to select the different histologic components of DFSP (DFSP center, DFSP infiltrative periphery, DFSP-T higher-grade sarcoma), in 22 patients followed over 3 to 156 months. EGFR protein and mRNA were expressed in 13/22 patients with DFSP or DFSP-T, and increased with tumor progression, both in microdissected areas of higher-grade sarcomas and in microdissected areas of local extension. No cancer-associated *EGFR* gene mutation or copy-number variation, nor any *KRAS, BRAF, NRAS* hotspot mutations were found in any microdissected area. Among epithelial-mesenchymal transition factors tested, SNAIL 1/2 had the same expression pattern as EGFR while ZEB1/2 or TWIST1/2 did not. Using a proteome profiler phospho-kinase array on 3 DFSP and 3 DFSP-T cryopreserved tissue samples, EGFR phosphorylation was detected in each case. Among EGFR downstream pathways, we found positive correlations between phosphorylation levels of EGFR and STAT5a/b (r = 0.87, *p* < 0.05) and TOR (r = 0.95, *p* < 0.01), but not ERK in the MAPK pathway (r = -0.18, *p* > 0.70). We thus demonstrated that in DFSP evolution to high grade sarcoma, EGFR and SNAIL were involved, with EGFR activation and signaling through TOR and STAT5a/b downstream effectors, which could lead on to new therapies for advanced DFSP.

## INTRODUCTION

Dermatofibrosarcoma protuberans (DFSP) accounts for 6% of all soft tissue sarcomas, and affects young and middle-aged adults. Its slow growth contrasts with a predisposition to local recurrence and evolution to higher-grade sarcoma, or “transformed DFSP” (DFSP-T) in 10-20% of cases [1]. Clinically, DFSP-T is characterized by rapid growth, shorter recurrence-free survival and greater metastatic potential than DFSP [2]. This heterogeneous clinical evolution is associated with a histologic heterogeneity. The histologic pattern of DFSP comprises bulky nodules with an infiltrative periphery of CD34^+^ fibroblastic spindle cells [3]. The evolution to DFSP-T is characterized by the occurrence of areas of atypical spindle or pleomorphic cells with numerous mitoses. These clinical and tissular heterogeneities could explain why the molecular mechanisms underlying tumor progression have not yet been deciphered and why no predictive biomarker is currently available.

Biologically, DFSP is characterized by a genomic rearrangement involving chromosomes 17 and 22, in a supernumerary ring chromosome, or in a reciprocal balanced translocation t(17;22)(q22;q13) [4]. This rearrangement places the *PDGFB* gene under the control of the constitutively active *COL1A1* promoter, leading to overexpression of *PDGFB,* and thus to sustained platelet-derived growth factor receptor (PDGFR) signaling as a result of an activating autocrine loop [4]. The first targeted therapy used in DFSP and DFSP-T was imatinib, a PDGFR tyrosine-kinase inhibitor. Response rates to imatinib do not exceed 50% using RECIST criteria and secondary resistance occurs among the responders, especially in metastatic DFSP and DFSP-T [5–9]. Therefore, other receptor tyrosine kinase pathways may be involved in DFSP progression.

Recently, epidermal growth factor receptor (EGFR) has been found phosphorylated in 7 patients with DFSP [7]. EGFR (also known as ERBB1 or HER1) is a cell-surface receptor tyrosine kinase whose ligands include epidermal growth factor and transforming growth factor-α [10]. EGFR signaling through receptor phosphorylation and activation of downstream effectors contributes to tumor cell proliferation, apoptosis evasion, angiogenesis and metastasis [11]. The EGFR downstream effectors include phospholipase C (PLC), Janus kinase (JAK)/Signal Transducer and Activator of Transcription (STAT), Mitogen-Activated Protein Kinase (MAPK), and phosphoinositide 3-kinase (PI3K)/protein kinase B (AKT)/Target of Rapamycin (TOR) pathways. Functional EGFR dysregulation, with overexpression and activation by mutations or autocrine/paracrine growth factor loops, has been identified in 50% of human epithelial malignancies, leading to EGFR-targeted therapy [12].

Here, we laser-microdissected areas from DFSP center, DFSP infiltrative periphery and DFSP-T higher-grade sarcoma in 22 patients followed over 3 to 156 months, and we assessed EGFR expression, mutational pattern, activation and signaling in each of them.

## RESULTS

Our study included 12 DFSP patients, mean age 42.3 ± 9.5 years with a male-to-female ratio of 1, and 10 DFSP-T patients, mean age 48 ± 20.2 years with a male-to-female ratio of 0.67 (Table 1). All 22 cases presented *COL1A1-PDGFB* rearrangement and none had received imatinib before tumor sampling.

**Table 1:** Clinical, biological and follow-up data of the 22 patients with DFSP or DFSP-T

Patient	Diagnosis	Age at diagnosis (years)	Sex	Tumor site	Largest Tumor size (cm)	Treatment	Follow-up duration (months)	Metastatic disease	Status at last follow-up
1	DFSP	54	M	Shoulder	2.5	Surgery	38	No	aned
2	DFSP	37	F	Thigh	2.5	Surgery	36	No	aned
3	DFSP	33	M	Thigh	2	Surgery	39	No	aned
4	DFSP	27	F	Chest	2	Surgery	38	No	aned
5	DFSP	50	F	Thigh	2	Surgery	40	No	aned
6	DFSP	40	M	Face	3	Surgery	26	No	aned
7	DFSP	55	F	Shoulder	1.5	Surgery	39	No	aned
8	DFSP	44	M	Chest	7	Surgery	10	No	aned
9	DFSP	41	F	Back	1.5	Surgery	42	No	aned
10	DFSP	47	M	Chest	3	Surgery	24	No	aned
11	DFSP	51	F	Arm	6.5	Surgery	39	No	aned
12	DFSP	29	M	Back	9	Surgery	156	No	aned
13	DFSP-T (FS)	21	M	Shoulder	2	Surgery	38	No	aned
14	DFSP-T (FS)	33	F	Thigh	4	Surgery	25	No	aned
15	DFSP-T (FS)	54	F	Abdomen	20	Imatinib, surgery and radiotherapy	110	No	aned
16	DFSP-T (FS)	48	M	Thigh	4.5	Surgery	24	No	aned
17	DFSP-T (FS)	23	F	Face	15	Imatinib	120	Yes	awd
18	DFSP-T (FS)	68	M	Abdomen	8	Surgery and radiotherapy	51	No	aned
19	DFSP-T (FS)	49	F	Back	6	Surgery	40	No	aned
20	DFSP-T (FS)	36	M	Thigh	9	Surgery	66	No	aned
21	DFSP-T (UPS)	83	F	Leg	17	Surgery	40	No	aned
22	DFSP-T (UPS)	65	F	Chest	16	Surgery	3	Yes	dod

Abbreviations: DFSP, dermatofibrosarcoma protuberans; DFSP-T, transformed dermatofibrosarcoma protuberans; FS, fibrosarcoma; UPS, undifferentiated pleomorphic sarcoma; M, male; F, female; aned, alive with no evidence of disease; awd, alive with disease; dod, dead of disease.

### EGFR is expressed in tumor cells from DFSP infiltrative areas and from DFSP-T higher-grade sarcoma areas

**Figure 1:**
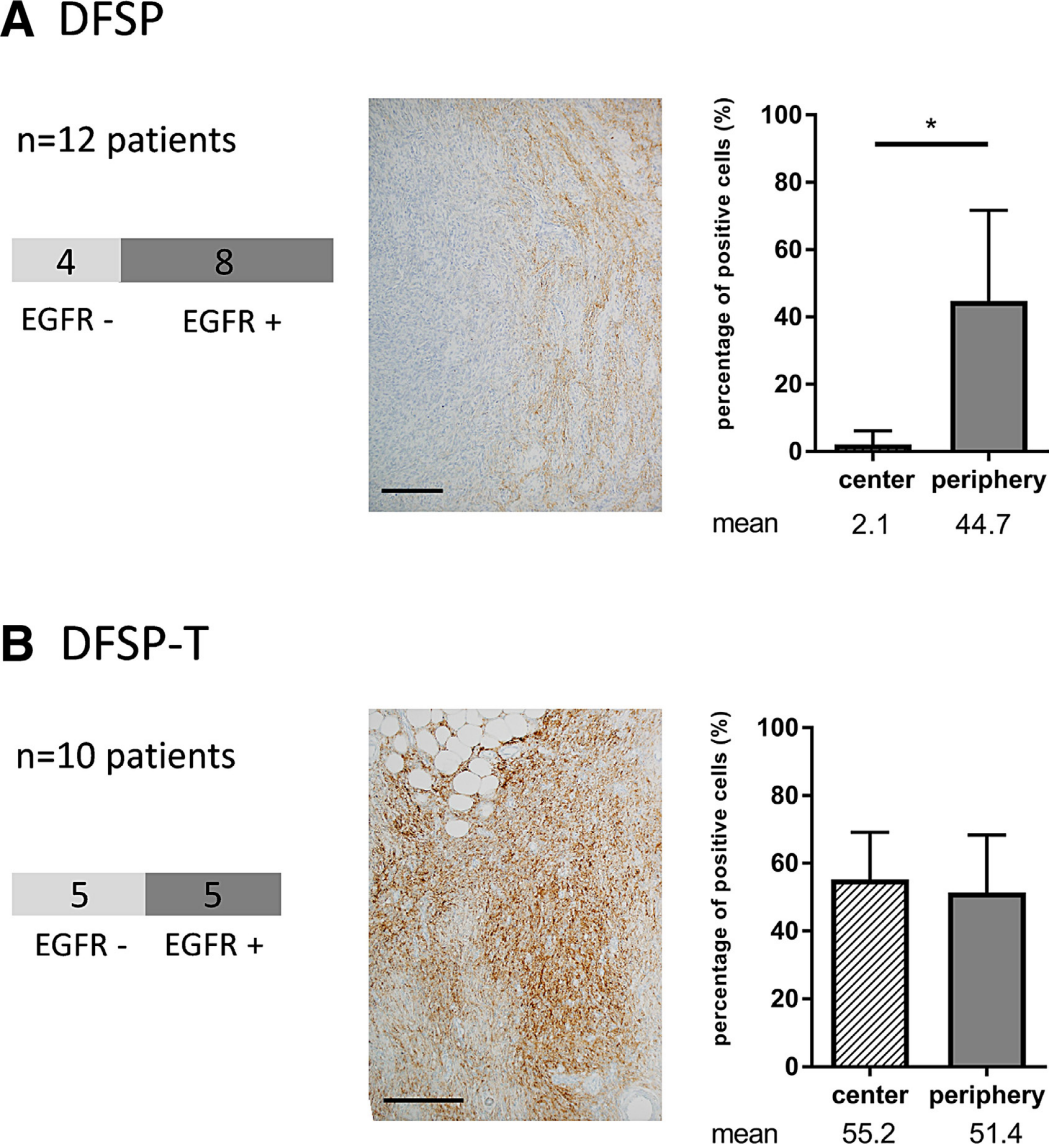
EGFR expression in patients with dermatofibrosarcoma protuberans (DFSP) and transformed DFSP (DFSP-T) (**A**) 8/12 DFSP patients have positive EGFR staining with a peripheral distribution of EGFR expression within the tumor; (**B**) 5/10 DFSP-T patients have positive EGFR staining within the tumor with a diffuse distribution of EGFR expression within the tumor. Scale bar = 100 μm; ^*^p < 0.05.

Among the 22 DFSP and DFSP-T, we first assessed EGFR expression using immunohistochemistry (Figure 1). EGFR expression was found in 8/12 DFSP (67%) and in 5/10 DFSP-T (50%).

Among the 13/22 patients with EGFR expression, when we analyzed the percentage of EGFR-positive tumor cells in the center and in the infiltrative periphery of the tumors, we found that, in DFSP, EGFR expression was significantly more marked in the peripheral invasive area than in the center of the tumor (44.7% vs 2.1%; *p* < 0.05). In addition, the percentages of EGFR-positive tumor cells were not different between DFSP-T and DFSP peripheral areas (*p* > 0.20).

**Figure 2:**
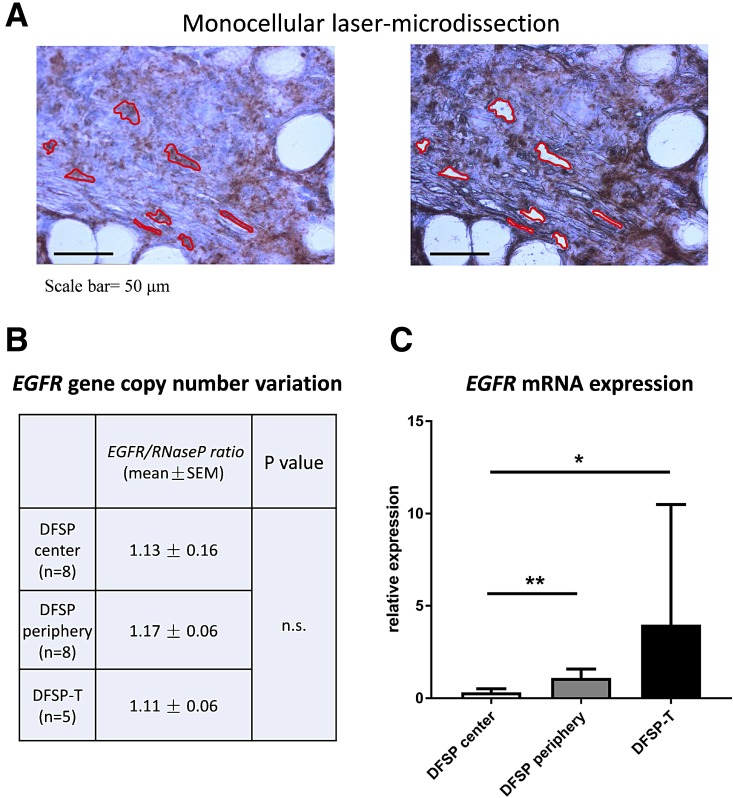
EGFR gene copy-number and mRNA analysis in microdissected tumor cells (**A**) monocellular tumor cell laser-microdissection was performed in DFSP center, DFSP periphery and DFSP-T samples (here represented DFSP periphery); (**B**) EGFR gene copy-number was in the normal range compared to Rnase P gene, in DFSP and DFSP-T; (**C**) EGFR mRNA expression increased significantly in DFSP periphery and DFSP-T compared to DFSP center (^*^p < 0.05; ^**^p <0 ,01).

The same pattern of expression, both on areas of local extension and on areas of higher-grade sarcoma, was found for *EGFR* mRNA in laser-microdissected cells (Figure 2): a significant increase was found in the DFSP infiltrative periphery compared to the DFSP center (1.11 ± 0.21 vs 0.32 ± 0.08; *p* < 0.01); the mean level of *EGFR* mRNA was highly elevated in DFSP-T, but no significant difference was observed between the DFSP-T and DFSP infiltrative periphery due to variation in *EGFR* mRNA expression among DFSP-T samples (DFSP-T, 3.98 ± 3.7 vs DFSP periphery, 1.11 ± 0.21; *p* > 0.20).

### The EGFR gene is not mutated in hotspots nor amplified in DFSP

We aimed to determine whether EGFR expression in the different microdissected areas was related to *EGFR* gene alterations, i.e. cancer hotspot point mutations, exon 19 deletion, or gene copy-number variations.

No L858R-activating mutation was found for the *EGFR* gene using allelic discrimination; no mutation or deletion was found on exons 18–21 using PCR-HRM and Sanger sequencing. Each microdissected sample was also tested for cancer hotspot mutations on *KRAS*, *BRAF* and *NRAS* genes, and for the T790M EGFR resistanc e mutation, but no mutation was found.

For the analysis of *EGFR* gene copy-number variation, we used quantitative droplet-digital PCR on DNA extracted from microdissected tumor cells (Figure 2B). In all DFSP and DFSP-T samples studied, the *EGFR/RNaseP* allele ratio was in the normal range, indicating no significant *EGFR* allele gain or loss in DFSP center, DFSP infiltrative periphery, or DFSP-T higher-grade sarcoma.

Overall, these results indicated that EGFR expression in DFSP and DFSP-T was not associated with known cancer-associated *EGFR* gene alterations, whether mutations or copy-number variations.

### EGFR and SNAIL are expressed in the same DFSP tumor areas

As EGFR expression in cancer may be linked to the epithelial-mesenchymal transition (EMT), and as EMT may impede the effect of EGFR-directed therapies [12], we analyzed the mRNA and protein expression of EMT factors in DFSP center, DFSP infiltrative periphery and DFSP-T higher-grade sarcoma areas.

**Figure 3:**
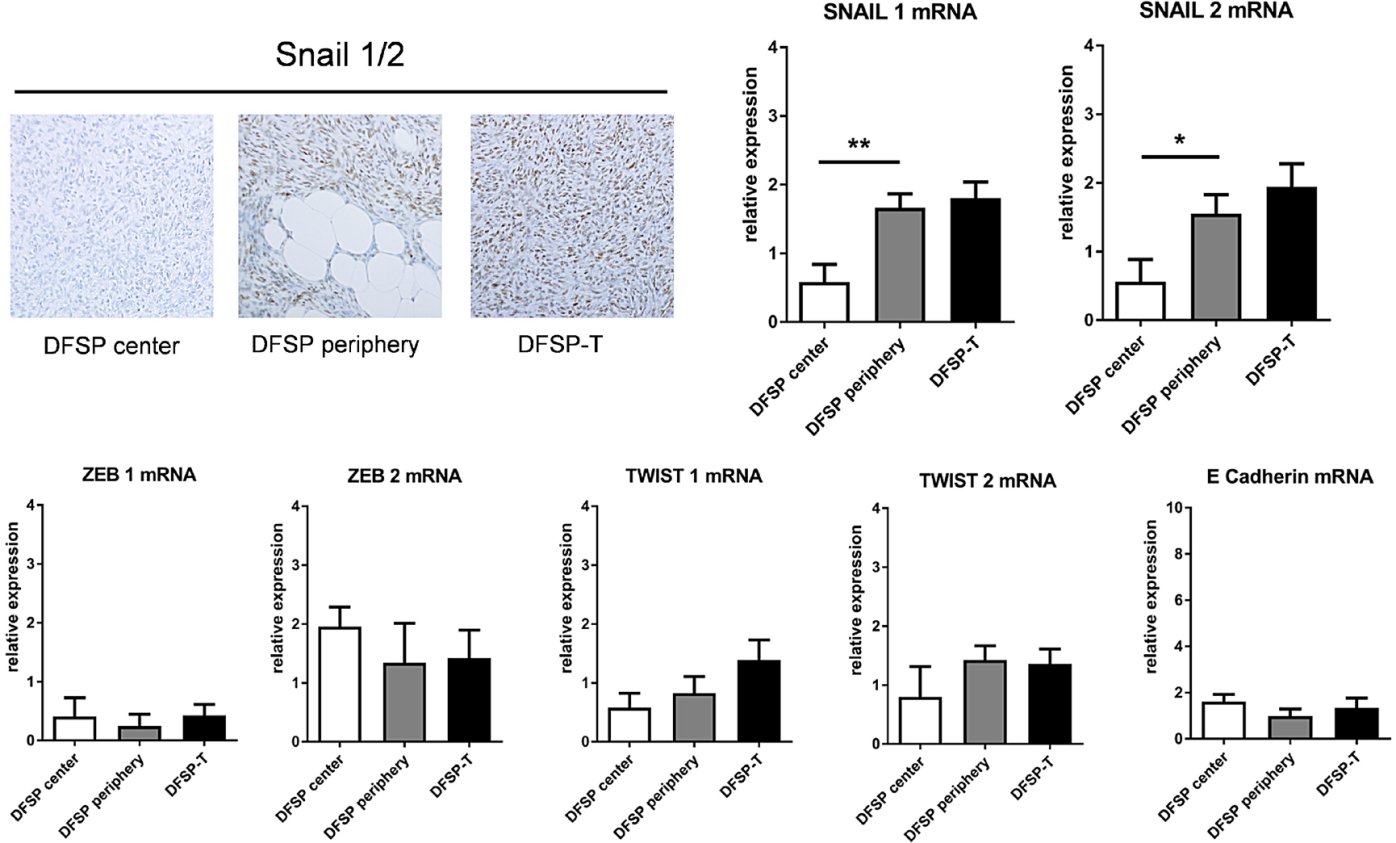
Expression of SNAIL and other epithelial-mesenchymal transition factors in patients with DFSP and DFSP-T The percentage of SNAIL1/2 positive cells on immunostaining increased in DFSP periphery and DFSP-T compared to DFSP center (upper left). For mRNA , SNAIL 1 and SNAIL 2 increased significantly in DFSP periphery and DFSPT compared to DFSP center (^*^p < 0.05; ^**^p < 0,01), whereas no significant difference was found for ZEB1, ZEB2, TWIST1, TWIST2 and E Cadherin.

A differential pattern of expression was only observed for SNAIL1 and SNAIL2, for both mRNA and protein (Figure 3). There was a significant increase in SNAIL protein expression in DFSP infiltrative periphery compared to DFSP center. When DFSP infiltrative periphery was compared to DFSP-T, there was no significant difference for mRNA (Figure 3), but the percentage of positive cells in immunohistochemistry was significantly higher in DFSP-T (66.8 ± 22% in DFSP periphery *vs* 90.3 ± 4% in DFSP-T areas; *p* < 0.05).

No difference in the patterns of expression was found for TWIST1, TWIST2, ZEB1, ZEB2, or E-Cadherin mRNAs in the 3 microdissected areas (Figure 3). No E-cadherin protein was detected using immunohistochemistry (data not shown).

Altogether, these results indicate that DFSP and DFSP-T had a molecular mesenchymal phenotype, and that no mesenchymal to epithelial transition occurred in DFSP and DFSP-T. In addition, SNAIL expression, as for EGFR, increased in areas of DFSP progression, especially DFSP-T.

### EGFR phosphorylation and downstream pathway activation in DFSP

We aimed to analyze whether EGFR expression in DFSP and DFSP-T was associated with EGFR activation through phosphorylation, and to determine which downstream signaling pathways were preferentially activated in DFSP tumor progression. For this, we used a proteome profiler phospho-kinase array on microdissected cryopreserved tissue samples of DFSP infiltrative periphery (*n* = 3) and DFSP-T higher-grade sarcoma (*n* =3 ).

EGFR phosphorylation was detected in all cases (mean relative quantity of phosphorylated protein compared to negative control, 2.45 ± 0.66). Among the potential downstream effectors of EGFR signaling, phosphorylation of ERK, STAT5a/b, and TOR was detected (2.60 ± 0.86; 2.52 ± 0.83; 1.87 ± 0.55 respectively), while PLCγ and STAT3 showed only slight phosphorylation (1.15 ± 0.03; 1.16 ± 0.13 respectively).

**Figure 4:**
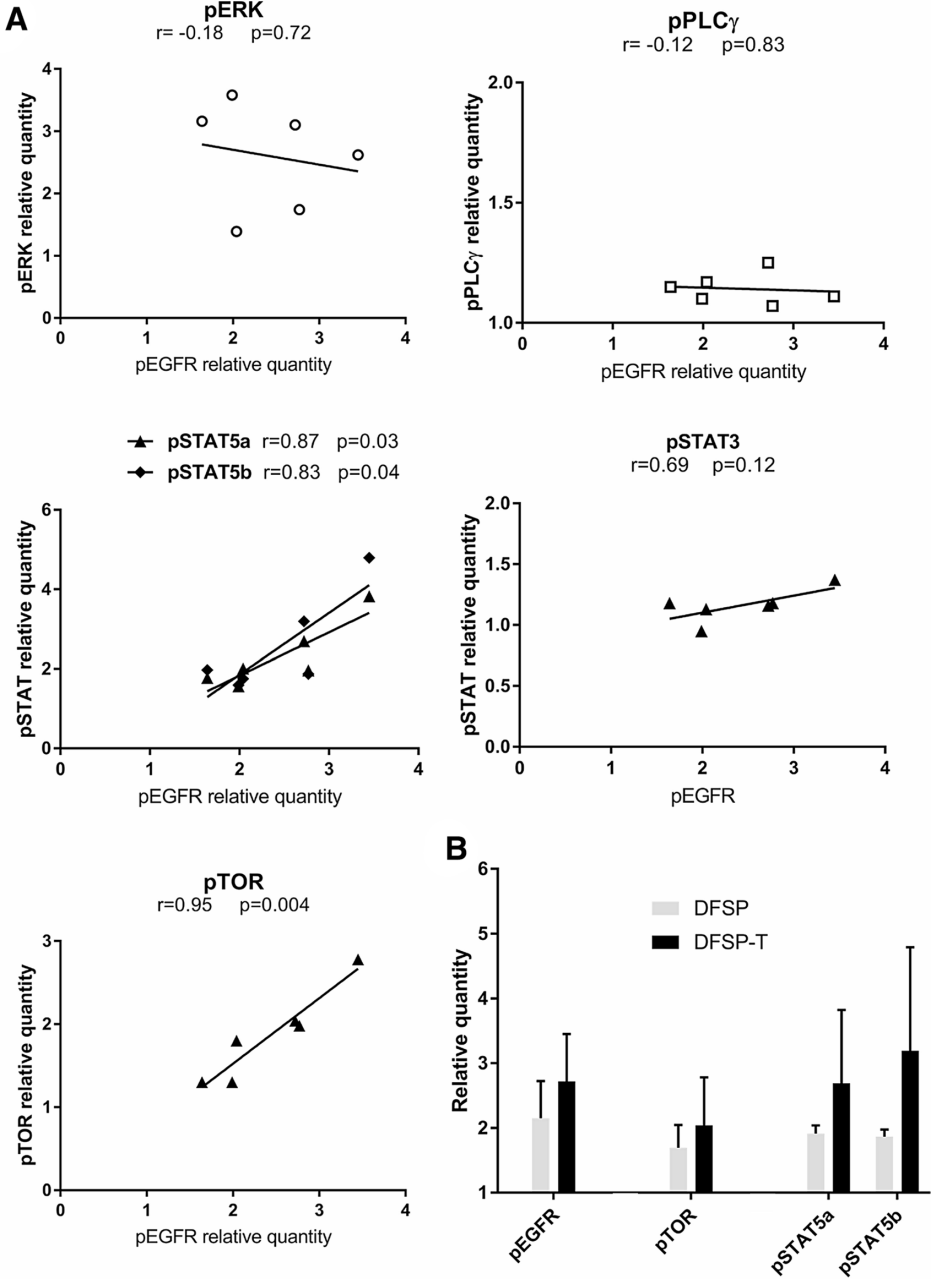
EGFR phosphorylation and downstream pathway activation in DFSP (**A**) The correlation analysis between the phosphorylation levels of ERK, PLCγ, STAT5a/b, STAT3 or TOR and the EGFR phosphorylation level showed a significant positive correlation for STAT5a/b and TOR. (**B**) The relative quantity of phosphorylated protein for EGFR, TOR, STAT5a, and STAT5b increased non-significantly in EGFR+ DFSP-T areas (*n* = 3) compared to EGFR+ DFSP areas (*n* = 3).

To analyze the possible signaling relationship between EGFR and ERK, STAT5a/b, STAT3, TOR or PLCγ, we studied the correlation of the phosphorylation levels of EGFR and that of each of these potential downstream effectors (Figure 4A). ERK, STAT3, and PLCγ phosphorylation levels were not correlated to EGFR phosphorylation levels. The phosphorylation levels of STAT5a/b and TOR were positively correlated to EGFR phosphorylation levels (r = 0.87/r = 0.83, *p* < 0.05; r = 0.95, *p* < 0.01, respectively).

When we quantified pEGFR, pTOR, and pSTAT5a/b in DFSP samples and DFSP-T samples, all phosphoprotein levels were higher in DFSP-T compared to DFSP (Figure 4B).

EGFR was thus activated in DFSP infiltrative periphery and DFSP-T, and EGFR signaling in DFSP tumor progression preferentially involved TOR and STAT5a/b downstream effectors, but not ERK in the MAPK pathway.

## DISCUSSION

We studied here the possible involvement of EGFR expression and signaling in DFSP evolution to high grade sarcoma, using laser-microdissection and tissue-based molecular methods in a series of 22 patients.

We first demonstrated that EGFR mRNA and protein are expressed by tumor cells in a majority of DFSP, preferentially in the areas of local extension and areas of higher-grade sarcoma transformation. Among the 12 previously reported series involving in all 1072 adult soft tissue sarcomas studied for EGFR expression, EGFR was frequently expressed in malignant fibrous histiocytomas, myxofibrosarcomas, synovial sarcomas and malignant peripheral sheath tumors, but few DFSP samples were tested [13–24]: 5 samples were negative in immunohistochemistry (IHC), and 4 others showed EGFR mRNA expression on Northern blot [14,22–24]. The discrepancy in IHC results between the 22 cases in our series and the 5 published cases could be linked to the different antibody clones used for EGFR detection: clone EGFR.113 in the studies by Dobashi *et al.* [14,24], known to detect a smaller number of positive cases [16], versus clone 31G7 in our study. One strength of our study was that it combined EGFR protein and mRNA detection in the same microdissected areas to provide more reliable results.

In 2005, high levels of EGFR expression in 281 patients with soft tissue sarcomas other than DFSP were significantly associated with the histological grade and with a shorter overall survival [17]. We also found in our 22 DFSP patients that EGFR expression increased with DFSP progression to high grade sarcoma, a known marker of poor prognosis [1, 2]. In our cohort, 2 patients with DFSP-T out of 10 developed metastasis (20%) and 1 died of disease (10%). These rates are in line with published data regarding DFSP evolution in large retrospective epidemiological cohorts: Liang CA *et al.* reported 14.4% metastasis and 14.7% death from disease in DFSP-T [1] ; Hoesly PM *et al.* reported 18% metastasis and 0% death from disease [2]. In our 2 DFSP-T patients with metastasis, EGFR expression was present. The low number of metastatic or death events in our cohorts precluded any statistical analysis including this variable. It will be of interest in the future to analyze further the prognostic value of EGFR expression for metastasis or death in DFSP.

We also demonstrated similar patterns for EGFR and SNAIL expression in the areas of DFSP progression to high grade sarcoma. In malignant fibrous histiocytomas, synovial sarcoma and osteosarcomas, SNAIL1 expression has been detected in areas of invasion, and correlated with the grade of the tumor [25,26]. In accordance, the evolution of DFSP to high grade sarcoma has been associated with a transcriptional reprogramming including epithelial-mesenchymal transition-like process, in 5 DFSP and 5 DFSP-T samples [27]. Previous studies have shown that the role of SNAIL is not restricted to triggering EMT in epithelial cells. In fibroblastic cell lines, SNAIL1 overexpression provided tumorigenic potential [28] and was required to drive the invasion of fibroblasts [29]. In soft tissue tumors, SNAIL1 may be expressed by cells with mesenchymal stem cells properties and higher metastatic potential [25]. As in cervical and gastric cancer, SNAIL expression in DFSP may be promoted by EGFR signaling [30–32].

In our 22 cases, as in 275 soft-tissue sarcomas reported elsewhere, EGFR expression was not linked to EGFR activating mutation [33], or to *EGFR* gene copy-number alteration. In other soft-tissue sarcomas, EGFR is activated through phosphorylation [14,17,19,34]. *EGFR* amplification is found only in 3.5 to 7% of soft-tissue sarcomas [14,16,24]. In our cases, laser microdissection enabled us to localize EGFR phosphorylation in the areas of DFSP local extension and DFSP-T higher-grade sarcoma. In these areas, EGFR phosphorylation was associated with downstream activation of TOR and STAT5a/b.

To date, few studies have focused on the pathways of EGFR signaling in soft-tissue tumors. As in our study, AKT/TOR pathway was reported to be predominantly activated in association with EGFR expression in 39 bone and soft-tissue tumors [14]. TOR activation was also found in 27 epithelioid sarcoma human samples and 2 cell lines expressing EGFR [19]. In EGFR-positive areas of our DFSP samples, the phosphorylation level of STAT5a/b was elevated. As an alternative cell survival pathway, STAT5a/b has a pro-tumor effect, via overexpression and activation, in head and neck squamous cell carcinomas [35], and EGFR has been shown to activate STAT5a/b in breast cancer cell lines [36–38]. In our DFSP patients, EGFR and STAT5a/b phosphorylation levels were correlated, suggesting that a similar STAT-mediated EGFR signaling could be involved DFSP evolution to high grade sarcoma. To validate our findings using functional studies, we attempted to establish patient derived xenografts of DFSP and DFSP-T primary tumors in nude mice, but the tumors did not engraft. We also attempted to develop primary cell culture from 2 DFSP primary tumor samples, but cells did not survive. In the literature, primary cell culture of DFSP-T was only achieved in one patient using metastatic imatinib-resistant DFSP-T tissue [5]. From our experience, it seems that DFSP or DFSP-T primary tumor biology is not adequate for patient derived xenograft or primary cell culture using conventional methods.

PDGFR-inhibition by imatinib provides clinical benefit in about 50% of DFSP patients, but secondary resistance occurs among the responders [5–9], and other therapeutic options are required. Our findings on EGFR expression, activation, and putative signaling through TOR and STAT5a/b in DFSP progression open fields for new therapeutic options in patients with non-operable or metastatic DFSP. The first test of EGFR inhibition using gefitinib and conventional chemotherapy was active *in vitro* and *in vivo* in a human fibrosarcoma cell lines [34], but a phase II clinical trial on gefitinib in EGFR-expressing *EGFR*-wild type advanced synovial sarcomas had low response rates and short-lived disease control [39]. Recently, preclinical studies combining EGFR blockade with either mTOR or STAT blockade overcame resistance to anti-EGFR monotherapy in EGFR-expressing soft tissue sarcomas and in fibrosarcoma cell line [19, 40].

In conclusion, using laser-microdissection and tissue-based molecular methods in 22 patients with DFSP, we found that EGFR was involved in DFSP progression to DFSP-T, associated with SNAIL overexpression, and mTOR and STAT5a/b signaling. These novel insights into the biology of DFSP tumor progression could help to optimize future clinical trials and could lead on to new targeted therapies for advanced DFSP.

## MATERIALS AND METHODS

### Patients and samples

Twenty-two patients with DFSP or transformed DFSP (DFSP-T) from a single university hospital (Hôpital Saint Louis) diagnosed between 2002 and 2015 were included in the study. They had enough formalin-fixed paraffin embedded and frozen tumor material remaining after the diagnosis had been established. All patients were informed that part of the remaining tissue material could be used for research, and gave their consent according to the declaration of Helsinki and to the French law. Samples were obtained from the Tumorothèque of the Hôpital Saint Louis. All samples were taken at diagnosis, before any medical treatment of the disease. Among the 22 patients, 12 had DFSP and 10 had DFSP-T (fibrosarcomatous or an undifferentiated pleomorphic variant) at diagnosis. Diagnoses were performed according to the latest 2013 WHO criteria, and validated in the French Sarcoma Pathology Network.

### *In situ* EGFR and mesenchymal-epithelial transition protein immunostaining

Immunohistochemical analyses were carried out on formalin-fixed paraffin-embedded (FFPE) tissue sections. An indirect immunoperoxidase method using anti-human EGFR mouse monoclonal antibody (clone 31G7, Abcam, Cambridge, UK) as the primary antibody was performed on 4 µm-thick tissue sections. Universal Secondary Antibody (Roche Diagnostics, Meylan, France) was used as the secondary antibody, and the DabMap kit (Roche Diagnostics) was used for detection. Appropriate controls with non-relevant isotype antibody and with omission of the primary antibody were implemented. Epidermal cells with a strong membrane expression of EGFR were used as a positive internal control.

Each section was examined at ×400 magnification on five randomly chosen fields in the central area of the tumor and five randomly chosen fields in the infiltrative periphery of the tumor. A ProvisBX51 light microscope (Olympus, Tokyo, Japan) was used, providing a field size of 0.344 mm^2^ at ×400 magnification. All samples were assessed independently by two investigators. The percentage of EGFR-expressing cells among all tumor cells in each field was calculated. Results were expressed as mean ± standard error of the mean (SEM).

The same method was used for the detection and quantification of Snail1/2 and E-cadherin expression. The primary antibodies were respectively anti-Snail/Slug rabbit polyclonal antibody (ab85936, 1/50 dilution, Abcam, Cambridge, UK) and anti-human E-cadherin mouse monoclonal antibody (clone HECD-1, 1/100 dilution, Abcam).

### Laser microdissection and nucleic acid extraction

Monocellular laser-microdissection of tumor cells from the EGFR-negative central DFSP area, the EGFR-positive peripheral invasive DFSP area, and the EGFR-positive higher-grade sarcoma DFSP-T area, was performed on 7-μm thick FFPE tissue sections using a Zeiss Microdissection and Pressure Catapulting system (Zeiss, Munich, Germany). A minimum of 1000 cells were microdissected for DNA studies for each area, corresponding to a minimum surface area of 228 000 µm^2^. Total DNA was extracted from the microdissected cells using DNeasy-Mini-Kit (Qiagen, Courtaboeuf, France).

For RNA studies, monocellular laser-microdissection was performed in the same areas, and total RNA was extracted from the laser-microdissected cells using miRNeasy kit (Qiagen). DNA and RNA were qualified and quantified using spectrometric assay (Nanodrop ND-1000, Thermo Scientific, Wilmington, USA).

### Cancer hotspot mutation analyses for EGFR, KRAS, NRAS and BRAF genes

Cancer hotspot mutation analyses were performed on microdissected cells from each area using: allelic discrimination method on an LC480 system (Roche) for *KRAS* G12S, G12R, G12C, G12D, G12A, G12V, G13D mutations, *EGFR* L858R, T790M mutations, *BRAF* V600E mutation; high-resolution melting (HRM) PCR mutation screening on LC480 system (Roche) for *KRAS* exons 2, 3, 4, *NRAS* exons 2,3, *EGFR* exons 18, 21, and *BRAF* exon 15; Sanger sequencing on an ABI3130 DNA sequencer (Applied Biosystems, Darmstadt, Germany) for each suspected mutation after HRM-PCR, and systematically for *EGFR* exons 19 and 20.

### Droplet-digital quantitative PCR for DNA copy number and RNA expression analyses

For *EGFR* gene copy number analyses on microdissected cells, the Droplet Digital Polymerase Chain Reaction (ddPCR) was performed using the QX100 ddPCR workflow system (Biorad, Hercules, CA, USA). The mix contained 20 ng of genomic DNA from microdissected cells, 10 µl of So Fast Eva Green Supermix (Bio Rad), 1 µl of *EGFR* probes (Hs00538812-cn, Life Technologies, Foster City, USA) and 1µl *RnaseP* probes (Taqman^®^ copy number Reference Assay, 4403326, Life Technologies) per well and the final volume for the reaction was 20 μl. Droplets were generated by a QX200 Droplet Generator (Biorad). PCR was carried out on the CFX96 Real Time System (Bio Rad). PCR was performed with an initial denaturing step at 95°C for 10mn, followed by 40 cycles of denaturing (95°C for 15s), and annealing (60°C for 1 mn). A post-amplification melting curve program was initiated by heating to 98°C for 10 mn and then cooling down to 12°C. Each PCR run included a no-template control. The results of ddPCR were generated using QX100 Droplet Reader (Biorad), and analysed using QuantaSoft software (Biorad). The ratio of *EGFR* positive droplets to *RnaseP* positive droplets was calculated for each sample. A ratio of 0.8–1.2 was considered as a normal copy number of the *EGFR* gene.

For RNA expression, quantitative analyses were carried out using a two-step reverse transcription ddPCR with the QX100 ddPCR workflow system (Biorad). Total RNA from the microdissected cells was converted into cDNA using the GoScript Reverse Transcription system (Promega, Madison, WI, USA). For the ddPCR, the following probes were used: EGFR (Hs01076090-m1), SNAIL1 (Hs00195591-m1), SNAIL2 (Hs00950344-m1), Twist 1 (Hs01675818-s1), Twist 2 (Hs02379973-s1), Zeb1 (Hs00232783-m1), Zeb2 (Hs00207691-m1), and E-Cadherin (Hs01023894, all from Life Technologies). Droplet generation and PCR amplification were carried out as described above. Expression levels were evaluated using the QuantaSoft software, comparing the number of positive droplets for the gene of interest to the number of positive droplets for the endogenous control gene TATA-Binding Protein (TBP, Hs00427620m1, Life Technologies), in each sample.

### Protein phosphorylation array analysis of EGFR and downstream signalling pathways

The phosphorylation of surface and intracellular proteins was studied using the Proteome Profiler Human Phospho-Kinase Array kit (ARY003B, R&D systems, Minneapolis, MN, USA) on laser microdissected cells from invasive areas of 3 DFSP frozen-tissue samples and higher-stage sarcoma areas of 3 DFSP-T frozen tissue samples.

Tumor lysate samples were centrifuged, and the supernatant placed in a new tube and used for further steps. The amount of lysate loaded into each array was 365µg. The array kit was run according to the product instructions. Pixel density analysis was performed on a 10min film exposure, providing the mean pixel density of each spot. The relative quantity of each phosphorylated protein was calculated from: mean pixel density of the phosphoprotein duplicate spots / mean pixel density of the PBS negative control duplicate spots.

### Statistics

Quantitative values were compared using Student’s *t*-test (two-tailed) or Pearson’s r-correlation test with the GraphPad Prism 7 software (GraphPad software, La Jolla, CA, USA). *P* values under 0.05 were considered significant. Results are displayed in bar graphs representing mean ± standard error of the mean (SEM) or as linear regressions for data for correlation studies.
